# Supplementary material for: DMRforPairs: identifying Differentially Methylated Regions between unique samples using array based methylation profiles
Source: BMC Bioinformatics. 2014 May 15;15:141. doi: 10.1186/1471-2105-15-141 (PMC4046028; doi:10.1186/1471-2105-15-141)
Supplement: Additional file 2 — DMRforPairs output for the comparison of A431-MCF7 and NA17018-NA17105. Please start from the HTML files in each folder. Available via the BMC Bioinformatics website. [file 1471-2105-15-141-S2.zip › 1394847754114233_MOESM2_ESM/A431_MCF7/figures/8597.pdf]

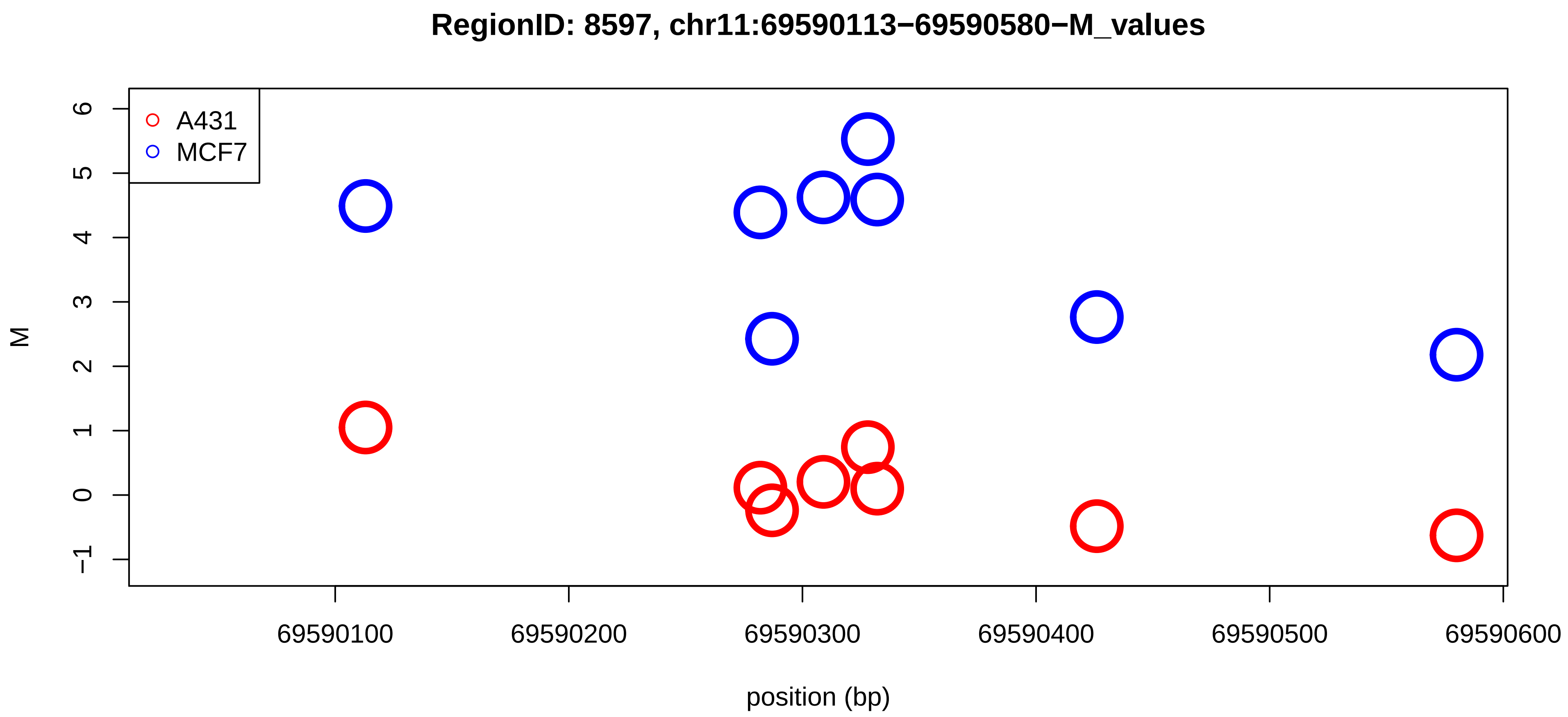
RegionID: 8597, chr11:69590113−69590580−M_values
A431
MCF7
M
−1
0
1
2
3
4
5
6
69590100
69590200
69590300
69590400
69590500
69590600
position (bp)

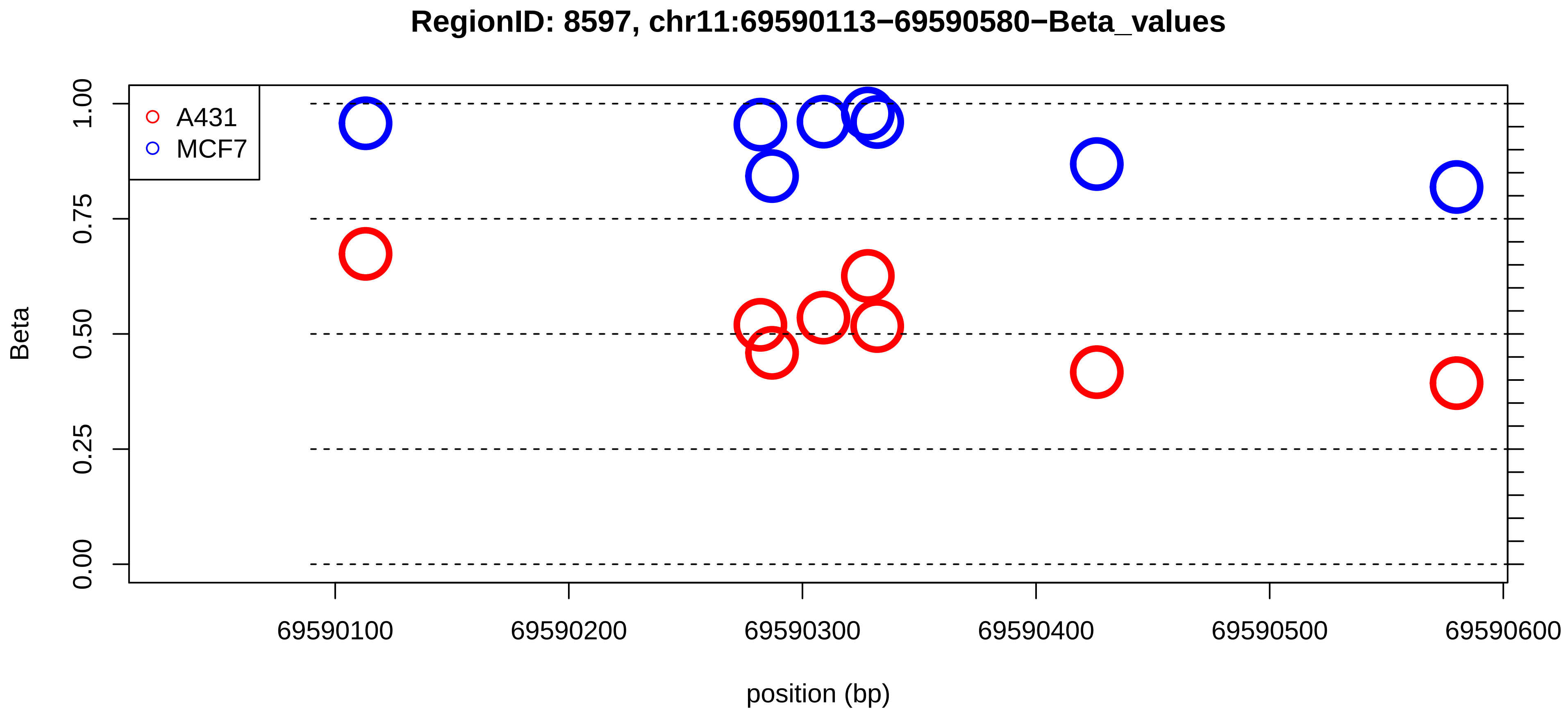
RegionID: 8597, chr11:69590113−69590580−Beta_values
A431
MCF7
Beta
0.00
0.25
0.50
0.75
1.00
69590100
69590200
69590300
69590400
69590500
69590600
position (bp)

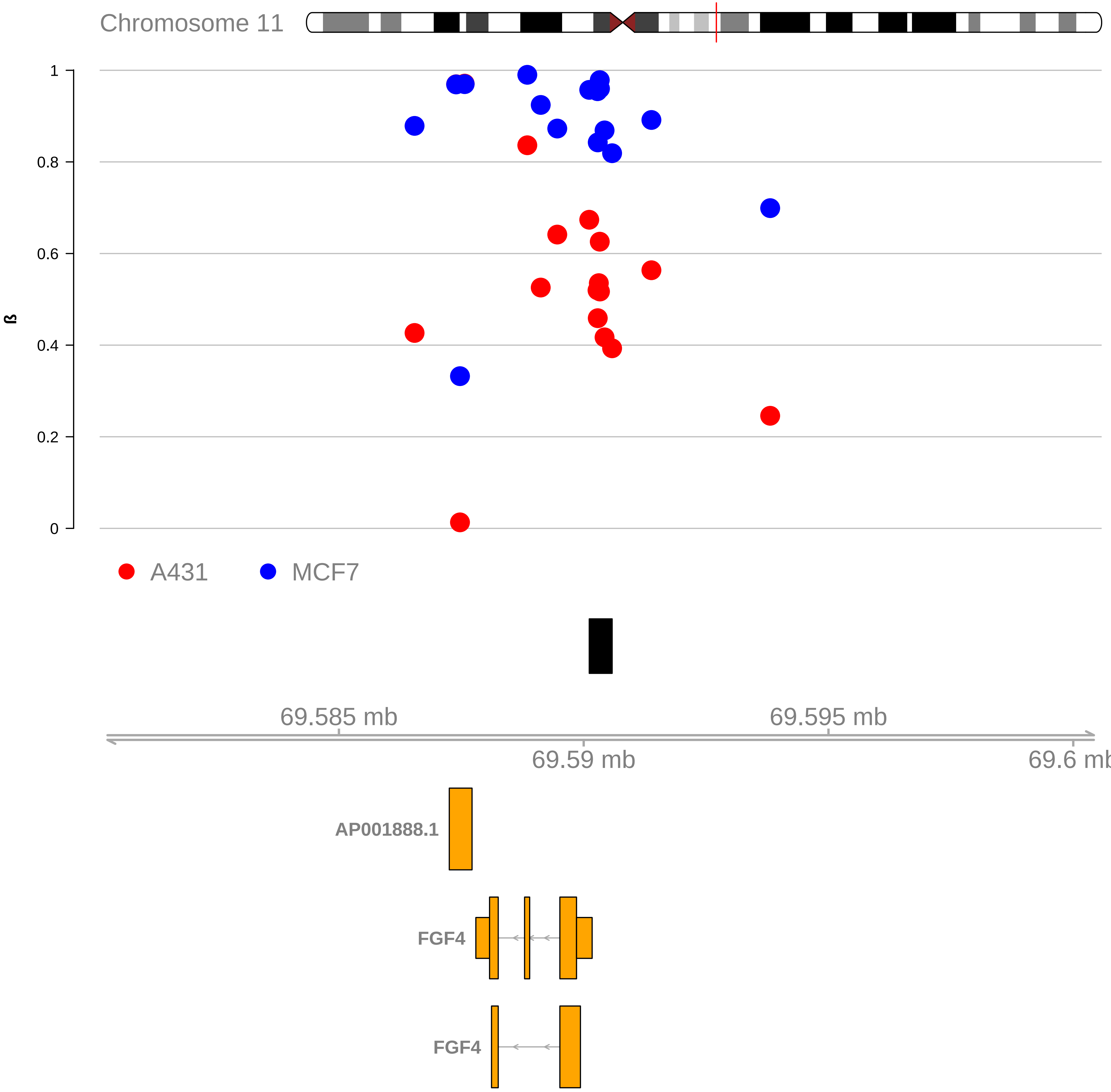
Chromosome 11
1
0.8
0.6
0.4
0.2
0
ß
A431
MCF7
69.585 mb
69.595 mb
69.59 mb
69.6 mb
AP001888.1
FGF4
FGF4
